# Physical growth, puberty and hormones in adolescents with Nodding Syndrome; a pilot study

**DOI:** 10.1186/1756-0500-7-858

**Published:** 2014-11-28

**Authors:** Theresa Piloya-Were, Beatrice Odongkara-Mpora, Hanifa Namusoke, Richard Idro

**Affiliations:** Department of Paediatrics and Child Health, Mulago Hospital/Makerere University College of Health Sciences, P.O Box 7072, Kampala, Uganda; Department of Paediatrics, Faculty of Medicine, Gulu University, Gulu, Uganda; Nuffield Department of Medicine, Centre for Tropical Medicine and Global Health, University of Oxford, Oxford, UK

**Keywords:** Nodding syndrome, Epilepsy, Growth, Puberty, Hormones

## Abstract

**Background:**

Nodding syndrome is an epidemic symptomatic generalized epilepsy syndrome of unknown cause in Eastern Africa. Some patients have extreme short stature. We hypothesized that growth failure in nodding syndrome is associated with specific endocrine dysfunctions. In this pilot study, we examined the relationship between serum hormone levels and stature, bone age and sexual development.

**Results:**

We recruited ten consecutive children, 13 years or older, with World Health Organization defined nodding syndrome and assessed physical growth, bone age, development of secondary sexual characteristics and serum hormone levels. Two children with incomplete results were excluded. Of the eight remaining, two had severe stunting (height for age Z [HAZ] score < -3) and three had moderate stunting (HAZ score between-3 and -2). The bone age was delayed by a median 3(range 0-4) years. Serum growth hormone levels were normal in all eight but the two patients with severe stunting and one with moderate stunting had low levels of Somatomedin C (Insulin like Growth Factor [IGF1]) and/or IGF binding protein 3 (IGFBP3), mediators of growth hormone function. A linear relationship was observed between serum IGF1 level and HAZ score. With the exception of one child, all were either pre-pubertal or in early puberty (Tanner stages 1 and 2) and in the seven, levels of the gonadotrophins (luteinising and follicle stimulating hormone) and the sex hormones (testosterone/oestrogen) were all within pre-pubertal ranges or ranges of early puberty. Thyroid function, prolactin, adrenal, and parathyroid hormone levels were all normal.

**Conclusions:**

Patients with nodding syndrome may have dysfunctions in the pituitary growth hormone and pituitary gonadal axes that manifest as stunted growth, delayed bone age and puberty. Studies are required to determine if such endocrine dysfunction is a primary manifestation of the disease or a secondary consequence of chronic ill health and malnutrition and if so, whether targeted interventions can improve outcome.

## Background

Nodding syndrome is an emerging and debilitating epidemic neurologic disorder that affects children and adolescents in parts of Eastern Africa [1–6]. There are an estimated 5000 - 10,000 affected children in the region [7]. This probably symptomatic generalized epilepsy syndrome is characterized by head nodding determined to be atonic seizures [5], and complicated by the development of other seizure types, cognitive and motor decline, malnutrition, behavior and psychiatric difficulties [4].The etiology remains unknown, however there has been an association with infestation by *Onchocerca volvulus,* the parasitic cause of river blindness [2, 3, 8, 9]. Current treatments are symptomatic and include seizure control with antiepileptic drugs, nutritional and physical therapy and rehabilitation and, management of psychiatric difficulties [7].

Several patients with nodding syndrome have extreme short stature and delayed development of secondary sexual characteristics [4]. The pathogenesis of this growth failure and delay in the onset of puberty are unknown. Similar short stature has been reported in Nakalanga syndrome: a syndrome with many physical characteristics similar to nodding syndrome. In particular, patients with Nakalanga syndrome had delayed development of secondary sexual characteristics, and very low height for age z-scores compared to control children [10–12]. We hypothesized that nodding syndrome is associated with specific endocrine dysfunctions and that such dysfunction may manifest with growth failure, delayed bone growth and puberty. In this pilot study, we assessed consecutive patients with nodding syndrome, measured serum hormone levels and examined the relationship between the hormone levels and stature, bone age and sexual development.

## Methods

### Design

This was a pilot cross sectional survey of hormone levels in Ugandan adolescents with nodding syndrome.

### Participants

Participants were consecutive patients aged 13 years and older with confirmed nodding syndrome as defined by the World Health Organization criteria [13] and attending follow up care in Kitgum General Hospital. All had a diagnostic EEG for seizure classification. Eight of the ten patients were part of the 22 who had had detailed clinical, neurophysiologic and brain imaging reported [4].

### Procedures

The study was approved by Makerere University School of Medicine Research and Ethics Committee. Parental consent was obtained but no assent was requested because of cognitive difficulties in the subjects.

#### Assessment of physical maturity and puberty

Details of the clinical assessment was reported earlier [4]. Sexual maturity was assessed using the Tanner Sexual Maturity Staging. Anthropometric measures were obtained; standing height was determined using a stadiometer and weight using an electronic weighing scale. The height for age, weight for height and weight for age Z scores were calculated using CDC 2000 standards. In addition, x-rays of the left wrist were performed to determine bone age and reported using a Greulich and Pyle Atlas [14]. Bone growth was considered delayed if it was 2 years lower than the chronological age.

#### Laboratory measurements

We drew 10 milliliters of early morning fasting blood to assess endocrine function. The endocrine tests were performed by a blinded technician (unaware of the patient group) in an independent laboratory. The tests included vitamin D, thyroid function (TSH, T3 and T4), growth hormone, IGF 1 and IGFBP3, epiandrosterone and cortisol, testosterone, prolactin, estradiol, follicle stimulating hormone and luteinising hormone. No stimulation tests were performed in this preliminary study. Assays for TSH, T3, T4, cortisol, DHEA, FSH, LH, estradiol, testosterone, vitamin D and parathyroid hormone were performed using the Cobas e411 analyser (Roche Diagnostics), a fully automated immunoassay. Levels of IGF1 and IGFBP3 were determined using an Enzyme linked immunosorbant assay (ELISA) Diagnostic system Laboratories Inc. (DSL). A level of IGF1 of 220 – 972 ng/ml and IGFBP3 of 3.3-10 μg/ml was considered normal; vitamin D levels of 30-100 ng/ml was sufficient, moderate deficiency was defined as 10-29 ng/ml and severe deficiency as <10 ng/ml.

### Data analysis

Children were categorised into three height for age (HAZ) z scores of < -3, -3 to -2 and > -2. The primary outcome measure was the proportion of patients with abnormal hormone levels in the different endocrine pathways in children with the three different HAZ score groups.

## Results

### Demographic features and general clinical characteristics

Ten patients were assessed. Two were excluded; the first had incomplete results and the second was later found to be younger than 13 years old. The median age of the remaining eight was 15 (range 13–18) years. Five were female and three, male. The median age at onset of the signs of nodding syndrome as observed by the family was 7 (range 5–9) years, the median duration of the signs at the time of the study was 8 (range 5–9) years and in all cases, the onset of signs was between the years 2002 – 2008.

Three patients were classified as having head nodding only while five had head nodding plus (head nodding and other seizure types) [2]. Prior to presentation, all had been on varying doses of Carbamazepine. Other previous anticonvulsants included Phenytoin and Phenobarbitone. There was no clear benefit with any of these treatments; thus, all were continuing to experience frequent head nodding and/or other seizures. In addition, all had moderate to severe cognitive impairment. Five had behavior and psychiatric difficulties including wandering, aggression, anxiety, and depression. One child had psychotic behavior. Table 1 summarizes the patient characteristics.

**Table 1:** **Demographic, Clinical and Hormonal Characteristics of 8 children with nodding syndrome**

ID	Age in years, gender	Duration of signs in years	Main clinical features	WAZ score	HAZ score	Tanner stage (Breast/Testis)	Bone Age (years)	Vit D(ng/ml)	IGF1(ng/ml)	IGFBP3(μg/ml)
1	15, Male	8	Head nodding only; Frequent head nods, triggered by food; has difficulties in speech	- 4.75	-4.04	2	10	21.3	157	2.8
2	14, Female	9	Head nodding only; head nods triggered by food or appearing spontaneously. Three siblings and cousins also have NS.	-1.85	-1.49	2	12	23.0	286	4.3
3	13, Male	8	Head nodding plus; Head nods appear spontaneously; Frequent hallucinations	-1.53	-2.63	1	9	21.9	295	4.1
4	15, Female	9	Head nodding plus; head nods triggered by food and cold weather. Aggressive behaviour and often disoriented.	-5.05	-3.07	2	Not done	33.7	216	3.8
5	18, Female	8	Head nodding plus; head nods triggered by food; periods of being aggressive; visual hallucinations	-1.11	-0.10	3	17	25.2	231	3.4
6	14, Female	5	Head nodding only; head nods triggered by food; 3 other siblings also have nodding syndrome.	-1.42	-1.78	2	12	25.8	332	4.6
7	13, Female	6	Head nodding plus; head nods are spontaneous; Severe burn injury with a submental fistula	-3.17	-2.62	2	10	19.3	268	3.9
8	13, Female	6	Head nodding plus; Head nods triggered by food and occur spontaneously. Severe and uncontrolled seizures, behavior difficulties and psychotic behavior	-3.22	-2.23	1	10	18.8	164	3.8
9**	15, Male	2	Head nodding only	-3.25	-3.05	1	Not done	34.6	No result	No result

HAZ score < -3 = severe stunting, HAZ score -3 to -2 = moderate stunting and HAZ > -2 = Normal. Vitamin D deficiency level < 30 ng/ml; Normal IGF1 = 220-972 ng/ml, IGFBP3 = 3.3-10 ug/ml Tanner staging was based on breast development for girls and testicular size for boys. **Excluded patient because of incomplete results.

### Physical growth, sexual maturity and endocrine function

**Figure 1:**
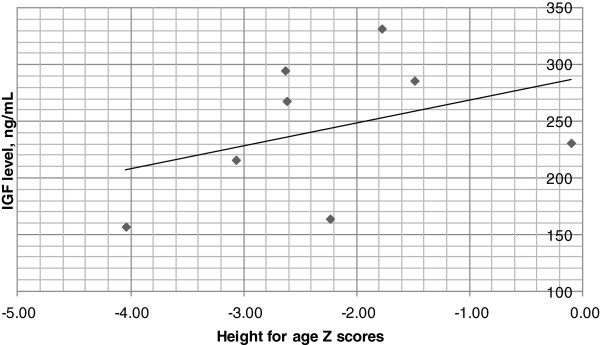
**Serum S-Somatomedin levels and Height for age Z scores in eight adolescents with nodding syndrome.** There is a linear relationship between the HAZ score and serum S-Somatomedin level.

Two of the eight patients had severe stunting (HAZ score < -3), three had moderate stunting (HAZ score -3 to -2) and the remaining three had normal height (HAZ score > -2). Compared to the chronologic age (median 14 [range 13-18] years), the bone age (median 11 [range 9-17] years) was delayed by a median 3 (range 0-4) years. Children with stunting had the most disparity in bone age. Vitamin D levels were low in seven of the eight patients with median 22.5 (range 18.8-33.7) ng/ml and all eight had osteopenia on x ray. The parathyroid hormone levels were however appropriate, Table 2.Growth hormone levels were normal (<5.4 ug/ml) in all eight patients, median 0.52 (range 0.12-1.06) ug/ml. The median Insulin like growth factor (IGF1) level was 250 (range 157- 332) ng/ml and the median IGF binding protein 3 (IGFBP3) level was 3.7 (range 2.8-4.6) μg/ml. Three patients (two with severe stunting and one of the three with moderate stunting) had low IGF1 levels. One of the two children with severe stunting also had low IGFBP3 level. IGF1 levels were however similar regardless of whether the child had head nodding only or head nodding plus (other seizures). Serum albumin levels were also normal in all the patients and there was no relationship between severe wasting and IGF1 or IGFBP3 levels. A linear relationship was however observed between serum IGF1 and the HAZ score, Figure 1.

**Table 2:** **Median (ranges) plasma levels of hormone in children with nodding syndrome**

Test (units)	Normal range	Median (range in patients
FT4 (pmol/l)	8.8 – 13.5	10.5 (8.8 – 20.1)
TSH (μIU/ml)	0.7 – 3.4	2.3 (1.27 – 3.18)
Cortisol (nmol/l)	171 - 536	256 (180 – 379)
DHEA (μmol/l	0.84 – 8.16	1.9 (0.84 – 5.24)
Parathyroid Hormone (pg/ml)	15 - 65	23.5 (16 - 65)
Prolactin (μg/l)	3.34 -26.71	5.75 (3.12 – 20)
FSH (IU/l)	**	4.4 (2.0 – 7.5)
LH (IU/l)	**	2.7 (1.5 – 7.9)
Estradiol (pmol/l)	**	169 (66 - 585)
Testosterone (nmol/l)	**	6.6 (5.2 -15.8)

**Normal limits of FSH, LH, testosterone and estradiol depend on sex and stage of sexual development.

Other than one child in Tanner Sexual Maturity Stage 3, all the other children were either pre-pubertal or in early stages of puberty. The profile of the gonadotrophins (luteinising and follicle stimulating hormone) and the sex hormones of testosterone/estrogen in these children further revealed values within the pre-pubertal or early stages of puberty. However, all eight patients had normal thyroid function with normal levels of thyroid stimulating hormone, T4 and T3. Adrenocorticotropic hormone and the adrenal corticosteroid and mineralocorticoid hormone levels were also all normal.

### Growth, age at onset of disease and hormone levels

Only one child had onset of symptoms before the age of five years. We therefore did not assess the effect of early onset of symptoms on growth or endocrine function. In addition, all remaining seven patients had similar duration of symptoms ranging from 5 – 8 years.

## Discussion

Nodding syndrome has been associated with growth failure. Like many other questions surrounding the syndrome, the mechanisms underlying the growth failure and delay in sexual development are unknown. This study explored if specific endocrine abnormalities in these patients was associated with the delayed growth and sexual development. The study found abnormal hormone levels in the pituitary-growth hormone axis, and in particular, low levels of IGF1 and IGFBP3 in patients with stunted growth, and delayed onset of puberty was associated with abnormal hormone levels in pituitary-gonadal axis and with low serum levels of the gonadotropins and sex hormones. In addition, almost all had low serum vitamin D levels. On the other hand, thyroid and adrenal hormone levels were normal.

### Implications of the findings

This is a pilot study based on only a handful of patients. Despite this limitation, the study suggests that endocrine dysfunctions in the growth hormone axis and the gonadal axis may be an important factor in the growth failure observed in adolescents with nodding syndrome. A more important question though is whether such dysfunction is a primary manifestation of the disease (for example, one that is mediated by an on-going inflammatory process) or is a secondary consequence of severe disease, chronic ill health and malnutrition. Psychological trauma from the longstanding rebel insurgency and the long period of suffering the community has experienced [15] may also have contributed to the suppression of the pituitary growth hormone and the gonadal axes. It is however unclear why the other axes including thyroid, parathyroid and adrenal hormones have remained normal. Understanding which of these pathways is responsible is likely to be important in targeting treatments. If endocrine dysfunction is secondary to severe disease, providing symptom control should result in correction of dysfunction and stimulation of growth. Furthermore, the mechanisms by which endocrine dysfunction develops could also be helpful in understanding the pathogenesis of nodding syndrome and bring us closer to determining etiology of the disease. Thus, a more detailed, larger and longer-term study is urgently required to examine which pathways is likely and may include growth hormone and gonadotropin stimulation tests.

Secondly, with a median 3 years of delayed bone age, there is room for catch up growth on current stature if, perhaps growth can be stimulated with symptomatic therapeutic interventions such as anticonvulsants and nutrition support or with more specific therapies such as treatment with growth hormone. However, with the long period of chronic ill health and malnutrition patients have experienced or disease processes possibly causing injury in the hypothalamic-pituitary areas, the most stunted patients are unlikely to achieve their target adult height despite the interventions.

The wide spread Vitamin D deficiency could be due to nutritional (dietary) deficiency, a side effect of prolonged use of the older anticonvulsants, the chronic debilitating disease or secondary to limited exposure to sunlight as patients remain hidden in houses due stigma. It is suggested that supplemental Vitamin D in patients with pharmaco-resistant epilepsy improves seizure control [16]. Therapeutic correction of Vitamin D deficiency should therefore be considered in nodding syndrome.

## Conclusions

Patients with nodding syndrome may have dysfunctions in the pituitary growth hormone and pituitary gonadal axes. These dysfunctions possibly manifest with stunted growth, delayed bone age and puberty. Studies are required to determine if this is a primary manifestation of the disease or a secondary consequence of chronic ill health and malnutrition and if so, whether targeted interventions can improve outcome.

### Availability of supporting data

All the data set supporting the results of this article is included within the main article.

## Authors’ information

Dr Theresa Piloya-Were (MBChB, MMED), a Paediatric Endocrinologist, is a lecturer in Makerere University College of Health Sciences. Dr Hanifa Namusoke (BSc, MSc [Human Dietetics], PhD) is a senior nutritionist in Mulago Hospital. Dr Beatrice Odongkara-Mpora (MBChB, MMED), is a Paediatric Endocrinologist and lecturer, Gulu University Faculty of Medicine. Dr Richard Idro (MBChB, MMED, PhD), is a Consultant Paediatrician/Paediatric Neurologist and Lecturer, Makerere University College of Health Sciences and Senior Clinical Research Paediatrician, Centre for Tropical Medicine and Global Health, University of Oxford.

## Abbreviations

FSH: Follicle stimulating hormone
LH: Luteinising hormone
GH: Growth hormone
IGF1: Insulin-like growth factor 1
IGF BP3: Insulin-like growth factor binding protein 3
T3: Tri iodo thyroxine
T4: Thyroxine.

## References

[1] Jilek LA (1964). Mental Diseases and Epilepsy in Tropical Africa. Fortschr Neurol Psychiatr Grenzgeb.

[2] Winkler AS, Friedrich K, Konig R, Meindl M, Helbok R, Unterberger I, Gotwald T, Dharsee J, Velicheti S, Kidunda A, Jilek-Aall L, Matuja W, Schmutzhard E (2008). The head nodding syndrome–clinical classification and possible causes. Epilepsia.

[3] Tumwine JK, Vandemaele K, Chungong S, Richer M, Anker M, Ayana Y, Opoka ML, Klaucke DN, Quarello A, Spencer PS (2012). Clinical and epidemiologic characteristics of nodding syndrome in Mundri County, southern Sudan. Afr Health Sci.

[4] Idro R, Opoka RO, Aanyu HT, Kakooza-Mwesige A, Piloya-Were T, Namusoke H, Musoke SB, Nalugya J, Bangirana P, Mwaka AD, White S, Chong K, Atai-Omoruto AD, Mworozi E, Nankunda J, Kiguli S, Aceng JR, Tumwine JK: **Nodding syndrome in Ugandan children–clinical features, brain imaging and complications: a case series.** *BMJ Open* 2013.,**3**(5)**:** 10.1136/bmjopen-2012-002540 PMC3646179 23645924

[5] Sejvar JJ, Kakooza AM, Foltz JL, Makumbi I, Atai-Omoruto AD, Malimbo M, Ndyomugyenyi R, Alexander LN, Abang B, Downing RG, Ehrenberg A, Guilliams K, Helmers S, Melstrom P, Olara D, Perlman S, Ratto J, Trevathan E, Winkler AS, Dowell SF, Lwamafa D (2013). Clinical, neurological, and electrophysiological features of nodding syndrome in Kitgum, Uganda: an observational case series. Lancet Neurol.

[6] Dowell SF, Sejvar JJ, Riek L, Vandemaele KA, Lamunu M, Kuesel AC, Schmutzhard E, Matuja W, Bunga S, Foltz J, Nutman TB, Winkler AS, Mbonye AK (2013). Nodding syndrome. Emerg Infect Dis.

[7] Idro R, Musubire KA, Byamah Mutamba B, Namusoke H, Muron J, Abbo C, Oriyabuzu R, Ssekyewa J, Okot C, Mwaka D, Ssebadduka P, Makumbi I, Opar B, Aceng JR, Mbonye AK (2013). Proposed guidelines for the management of nodding syndrome. Afr Health Sci.

[8] Foltz JL, Makumbi I, Sejvar JJ, Malimbo M, Ndyomugyenyi R, Atai-Omoruto AD, Alexander LN, Abang B, Melstrom P, Kakooza AM, Olara D, Downing RG, Nutman TB, Dowell SF, Lwamafa DK (2013). An Epidemiologic Investigation of Potential Risk Factors for Nodding Syndrome in Kitgum District. Uganda PLoS One.

[9] Spencer PS, Vandemaele K, Richer M, Palmer VS, Chungong S, Anker M, Ayana Y, Opoka ML, Klaucke BN, Quarello A, Tumwine JK (2013). Nodding syndrome in Mundri county, South Sudan: environmental, nutritional and infectious factors. Afr Health Sci.

[10] Jelliffe DB, Jones PR, Stroud CE (1962). Nakalanga notes on the endemic dwarfism of Uganda. Trop Geogr Med.

[11] Kipp W, Burnham G, Bamuhiiga J, Leichsenring M (1996). The Nakalanga syndrome in Kabarole District, Western Uganda. Am J Trop Med Hyg.

[12] Marshall AJ, Cherry JK (1961). Endocrine dysfunction in a Nakalanga dwarf. Trans R Soc Trop Med Hyg.

[13] WHO (2012). A report of the International Scientific Meeting on Nodding Syndrome.

[14] Acheson RM, Fowler G, Fry EI, Janes M, Koski K, Urbano P, Werfftenboschjj VA (1963). Studies in the Reliability of Assessing Skeletal Maturity from X-Rays. I. Greulich-Pyle Atlas. Hum Biol.

[15] McMullen JD, O’Callaghan PS, Richards JA, Eakin JG, Rafferty H (2012). Screening for traumatic exposure and psychological distress among war-affected adolescents in post-conflict northern Uganda. Soc Psychiatry Psychiatr Epidemiol.

[16] Hollo A, Clemens Z, Kamondi A, Lakatos P, Szucs A (2012). Correction of vitamin D deficiency improves seizure control in epilepsy: a pilot study. Epilepsy Behav.

